# Evaluation of the corneal topography based on deep learning

**DOI:** 10.3389/fmed.2023.1264659

**Published:** 2024-01-04

**Authors:** Shuai Xu, Xiaoyan Yang, Shuxian Zhang, Xuan Zheng, Fang Zheng, Yin Liu, Hanyu Zhang, Lihua Li, Qing Ye

**Affiliations:** ^1^Key Laboratory of Weak-Light Nonlinear Photonics, Ministry of Education, School of Physics and TEDA Applied Physics, Nankai University, Tianjin, China; ^2^Tianjin Eye Hospital, Tianjin, China; ^3^Tianjin Key Lab of Ophthalmology and Visual Science, Tianjin, China; ^4^Nankai University Affiliated Eye Hospital, Tianjin, China; ^5^Eye Hospital Optometric Center, Tianjin, China; ^6^School of Medicine, Nankai University, Tianjin, China

**Keywords:** deep learning, image processing, corneal topography, orthokeratology lens, treatment zone

## Abstract

**Purpose:**

The current study designed a unique type of corneal topography evaluation method based on deep learning and traditional image processing algorithms. The type of corneal topography of patients was evaluated through the segmentation of important medical zones and the calculation of relevant medical indicators of orthokeratology (OK) lenses.

**Methods:**

The clinical data of 1,302 myopic subjects was collected retrospectively. A series of neural network-based U-Net was used to segment the pupil and the treatment zone in the corneal topography, and the decentration, effective defocusing contact range, and other indicators were calculated according to the image processing algorithm. The type of corneal topography was evaluated according to the evaluation criteria given by the optometrist. Finally, the method described in this article was used to evaluate the type of corneal topography and compare it with the type classified by the optometrist.

**Results:**

When the important medical zones in the corneal topography were segmented, the precision and recall of the treatment zone reached 0.9587 and 0.9459, respectively, and the precision and recall of the pupil reached 0.9771 and 0.9712. Finally, the method described in this article was used to evaluate the type of corneal topography. When the reviewed findings based on deep learning and image processing algorithms were compared to the type of corneal topography marked by the professional optometrist, they demonstrated high accuracy with more than 98%.

**Conclusion:**

The current study provided an effective and accurate deep learning algorithm to evaluate the type of corneal topography. The deep learning algorithm played an auxiliary role in the OK lens fitting, which could help optometrists select the parameters of OK lenses effectively.

## 1 Introduction

In recent decades, the prevalence of myopia has increased dramatically worldwide, with a trend toward affecting younger ages (1, 2). It has been documented that the prevalence of myopia in children and adolescents in China will be 84% in 2050 (3). Myopia has been suggested to be a great burden on society in both the economic and public health systems (4).

Many optical interventions have been studied to retard myopia progression, such as multifocal contact lenses (5), multifocal spectacle lenses, and orthokeratology (OK) lenses (6–8). OK lenses have shown one of the greatest myopia control effects among these optical methods, with the slowing axial elongation ranging from 32% to 63% (9).

Corneal topography is an indispensable measurement in pre-treatment screening (10) and evaluating lens performance (11–14). The color of the topography interprets the morphological patterns (15), with different colors reflecting the variety of corneal diopter power. In the previous studies, usually, the basic information of corneal topography, such as flat K, steep K, and E value, were studied (16, 17), while few of them investigated the decentration, treatment zone, and pupil (18). For example, it has been reported that decentration is negatively correlated with axial elongation in myopic children (19, 20). Currently, two methods are used in corneal topography classification in clinics. One is based on the modeled force acting on corneal shape changes, such as bull's-eye, smiley-face, central islands, and corneal astigmatism. The other way is based on the decentration distance, which is classified as mild decentration (< 0.5 mm), medium decentration (0.5–1 mm), and severe decentration (>1.0 mm) (21). However, the methods mentioned above were proposed according to the changes in corneal shape. They were not connected with the defocus power of the OK lens, which may not predict the myopia control effects. Therefore, we proposed a new classification for corneal topography according to the effective defocusing contact range.

Deep learning plays a powerful role in the analysis of corneal topography, (22, 23) and it can learn various semantic information in images by building deep neural networks (24). Deep learning has been used to classify corneal topography between keratoconus and normal corneas because of the significant difference in corneal shape between them, and neural networks could easily extract image features (22, 25). However, it is difficult to accurately distinguish the corneal topographical characteristics among normal corneas using deep neural networks due to the insignificant difference in corneal shapes among them. In the past, semantic segmentation was used to identify the content and location of objects in the image (26, 27). In the current study, we aimed to use semantic segmentation to identify the important medical zones in corneal topography. In this study, semantic segmentation of the pupil and treatment zone is performed based on deep learning (28). We then calculate the medical indicators of decentration and effective defocusing contact range and finally evaluate the type of corneal terrain according to the evaluation criteria given by the optometrists.

## 2 Materials and methods

### 2.1 Data collection method

This study was approved by the Ethics Committee of the Tianjin Eye Hospital Optometric Center (ID 2023003) and adhered to the tenets of the Declaration of Helsinki. All participants provided informed consent. A Tomey corneal topographer (Takaratomy, Japan) was used to measure the corneal topography (29). The data of 1,302 myopic children and adolescents, aged 6–18 years, in the Tianjin Eye Hospital Optometry Center from 2013 to 2021 were collected retrospectively (2,604 eyes in total). Among them, data from 500 eyes that completed corneal topography were used for statistical and artificial intelligence analysis. The corneal topographic data were collected at baseline and after 1 month of OK lens wear. The specific operations were as follows. The collected images were exported in the form of tangent images through the automated scripts and corneal topography software (TMS-4A SW). Patients with previous experience with OK lens wear, poor fitting performance, and corneal diseases were excluded (30).

### 2.2 Materials

**Figure 1:**
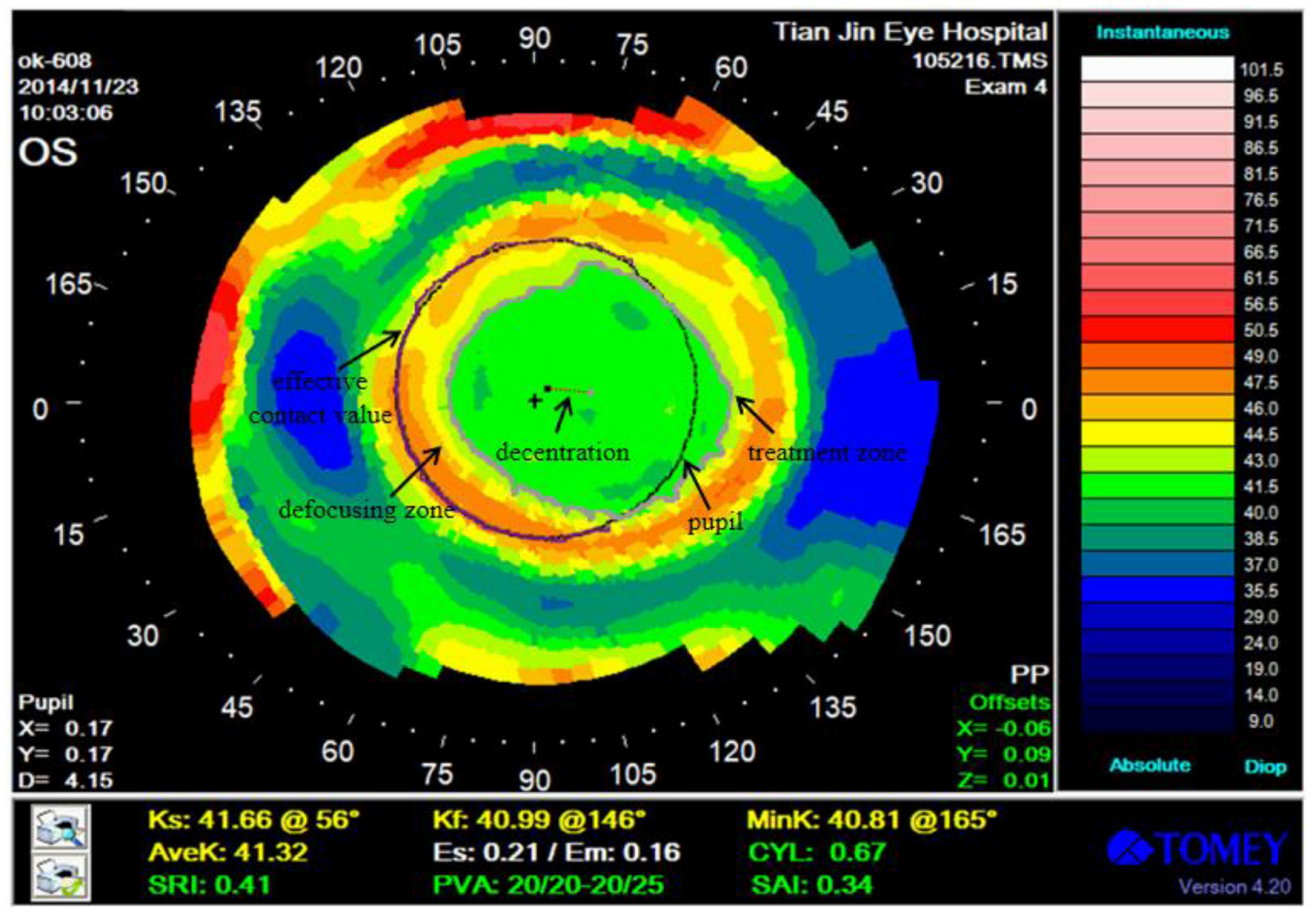
Corneal topography after 1 month of OK lens wear. Treatment zone, pupil, decentration, and effective defocusing contact range are marked in the figure.

The corneal topography was measured after 1 month of lens wear, which reflected the corneal characteristics (Figure 1). We determined the different zones of corneal topography and pupil as follows for analyzing the OK lens performance.

Treatment zone: the zone of the corneal surface that provides functional vision. It is the zone with the smallest central corneal aberration and the best visual quality.

Pupil: the hole in the middle of the iris, which refers to the circle of black pixels in the middle of the corneal topography.

Decentration: the distance between the pupil and the center of the treatment zone.

Effective defocusing contact range: the intersection of the defocusing zone boundary and the pupil boundary is the effective contact value, and the proportion of the effective contact value in the pupil boundary is the effective defocusing contact range.

### 2.3 Research framework

**Figure 2:**
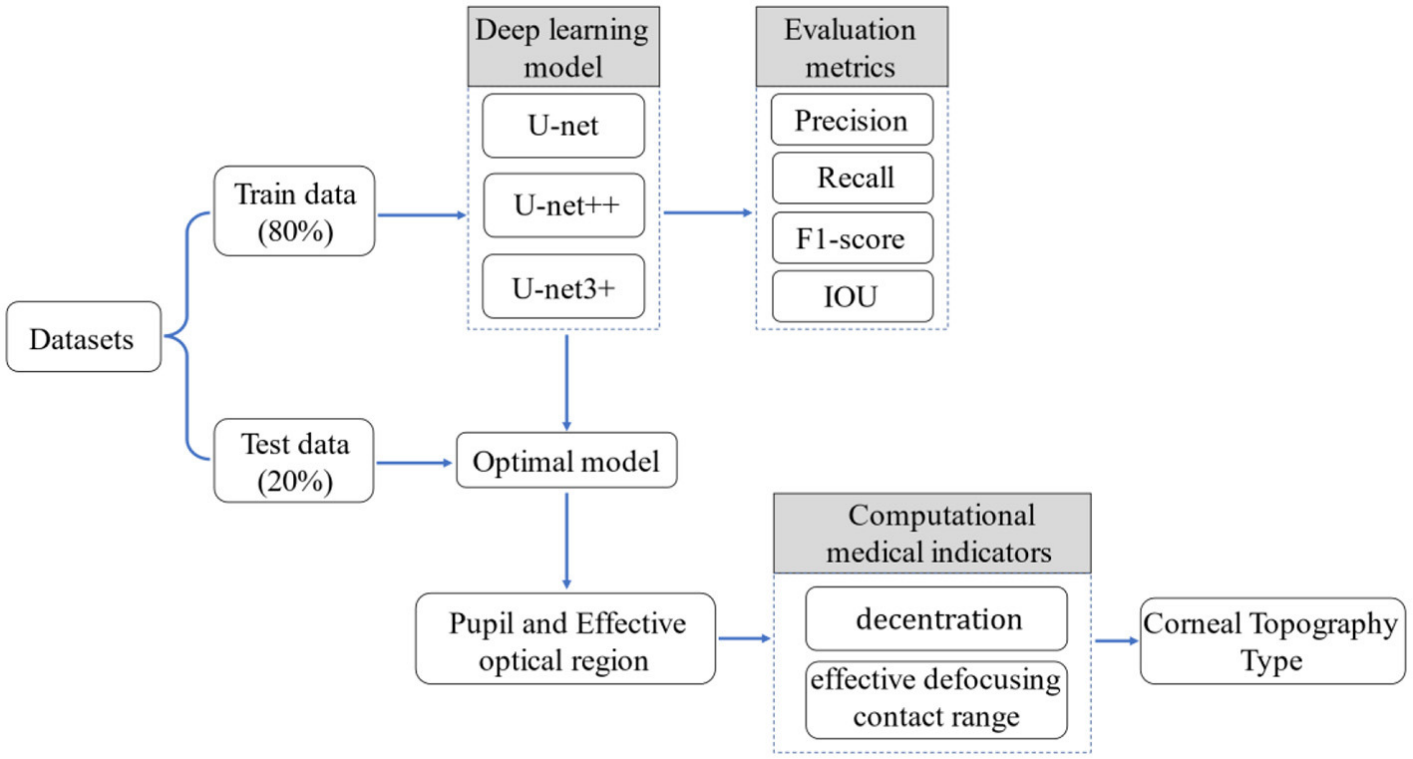
Research framework process.

In the current study, a unique type of corneal topography evaluation method based on deep learning and traditional image processing algorithms (Figure 2) was proposed. The treatment zone and pupil in corneal topography were segmented by the U-Net series of neural networks (31, 32). Decentration and effective defocusing contact range in the corneal topography was defined by an experienced optometrist, and a variety of image processing algorithms were used for calculation. Finally, the types of corneal topography were evaluated by combining the evaluation criteria provided by the optometrist with the results of various medical indicators (decentration and effective defocusing contact range).

### 2.4 Important medical zones

#### 2.4.1 Segmentation method

**Figure 3:**
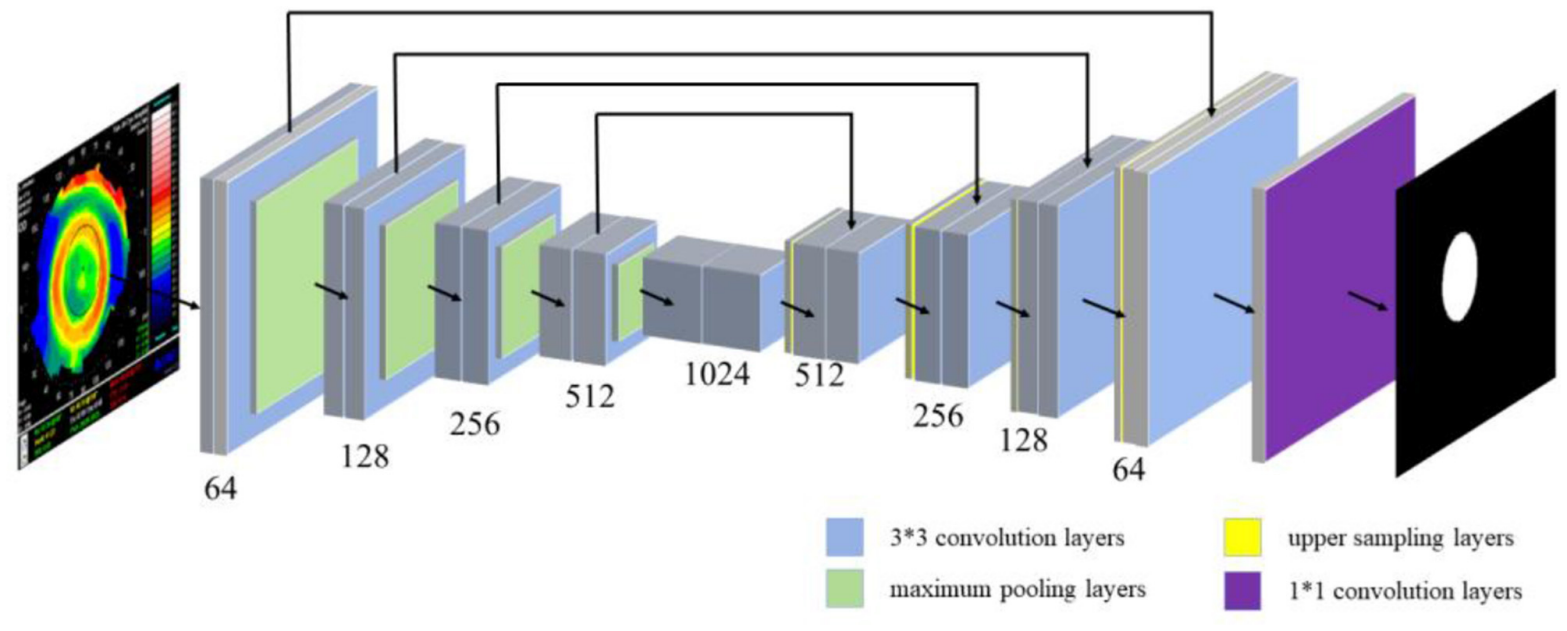
U-Net network structure.

The location information of the pupil and the treatment zone in the corneal topography were segmented by semantic segmentation. The semantic segmentation was usually interpreted as the classification of pixels in an image, so a U-Net series of neural networks was proposed to solve the problem (semantic segmentation of important medical zones). The jump connection in U-Net helps the convergence of the deep network and prevents the gradient from disappearing in the training process. The U-Net network structure is shown in Figure 3, including 19 3^*^3 convolution layers (blue rectangle), four maximum pooling layers (green rectangle), 4 upper sampling layers (yellow rectangle), and 1 1^*^1 convolution layer (purple rectangle). U-Net++ introduces deep supervision and multi-scale skip connections to improve network structure. It is worth noting that U-Net++ integrates feature maps from specific decoder and encoder layers, achieving a more robust semantic segmentation process through feature superposition. On this basis, U-Net3+ incorporates full-scale skip links, allowing for the fusion of feature maps across every decoder and encoder layer. This comprehensive integration facilitates a more holistic understanding of the input data. Additionally, U-Net3+ introduces a classification-guided module, which plays a vital role in ensuring precise segmentation. Therefore, 809 corneal topographies with good quality (the image acquisition is complete and the corresponding patient has no eye disease) were selected, and the specific zones of the pupil and treatment zone were manually marked with labeling software under the guidance of the optometrist (33) to facilitate semantic segmentation in the subsequent use of the U-Net neural network. To ensure the accuracy of the label, we asked two ophthalmic optical directors to recheck the label results during the labeling of the original data.

In the design of the loss function, the cross-entropy loss function was used (33). Suppose there are N pixels in total; the true annotation is expressed as r_n_, and the predicted probability is expressed as p_n_, so the cross entropy (CE) is described by Eq. 1:


(1) $$\begin{array}{l} \text{CE}=-\frac{1}{\text{N}}\overset{\text{N}}{\underset{\text{n}=1}{\sum }}{\text{r}}_{\text{n}}\log{\text{p}}_{\text{n}}+\left(1-{\text{r}}_{\text{n}}\right)\log\left(1-{\text{p}}_{\text{n}}\right) \end{array}$$

Precision, recall, f1 score, and Intersection over Union (IoU) in the segmentation model were selected to evaluate segmentation performance (Eq. 2–5):


(2) $$\begin{array}{l} \text{Precision}=\frac{\text{Truth Positive}}{\text{Truth Positive }+\text{ False Positive}} \end{array}$$

(3) $$\begin{array}{l} \text{Recall}=\frac{\text{Truth Positive}}{\text{Truth Positive }+\text{ False Negative}} \end{array}$$

(4) $$\begin{array}{l} \text{F}1=2\;\frac{\text{PrecisionRecall}}{\text{Precision}+\text{Recall}} \end{array}$$

(5) $$\begin{array}{l} \text{IOU}=\frac{\text{Ground Truth }\cap \text{ Detection Box}}{\text{Ground Truth }\cup \text{ Detection Box}} \end{array}$$

#### 2.4.2 Training strategy

The original images and their corresponding labels were input into the neural network together. They randomly selected 80% as the training set to train the semantic segmentation model and the other 20% as the test set to test the performance of the model. To achieve faster convergence in the training phase, the Adam optimizer was used for gradient descent (34). In terms of setting experimental parameters, the selection of learning rate and batch size is a key factor in model performance and training stability. The learning rate refers to the step size of each parameter update. An excessive learning rate may lead to unstable training, and the model cannot achieve minimum loss. A low learning rate can lead to slow convergence speed and may fall into local minima. Batch size refers to the size of the data batches used in each training session. An excessive batch size may lead to underfitting of the model, while an excessively low batch size may prolong training time and may not perform as well as models with larger batch sizes. In the experiment, our initial learning rate was selected as 10^−3^, 10^−4^, and 10^−5^, while the batch size was selected as 16, 32, and 64. We trained using the grid search method and ultimately set the initial learning rate to 10^−4^ and the batch size to 32. At the same time, all input images were resized to 224 × 224 and trained for 100 epochs to obtain the optimal results. Based on the loaded pre-training model, the semantic segmentation task was realized through transfer learning. In the test stage, the corneal topography was enhanced by rotation, clipping, and adding noise, which is used as the input of the model, and the pupillary and treatment zone segmentation effect images were obtained. The whole program was implemented based on the Pytorch deep learning framework.

### 2.5 Calculation method of the medical indicators

Treatment zone, pupil, decentration, and effective defocusing contact range were calculated based on the OpenCV library (35).

The decentration was calculated as the Euclidean distance between the pupil and treatment zone after obtaining the position information of the pupil and the treatment zone. The formula is shown in Eq. 6, where x and y are the coordinates of the corresponding center point zone, respectively.


(6) $$\begin{array}{l} \text{Decentration}=\sqrt{{\left({\text{x}1}^{2}-{\text{x}2}^{2}\right)}^{2}+{\left({\text{y}1}^{2}-{\text{y}2}^{2}\right)}^{2}} \end{array}$$

Decentration represented the distance between the center point of the pupil and the treatment zone. To obtain this indicator, the following operations should be carried out: (1) The pupil and the treatment zone were segmented using the above-trained U-Net3+ model. (2) The contour of the segmented zone was detected, and the coordinates of the center point were located by its circumscribed rectangle. (3) The distance between the pupil and the center point of the treatment zone is calculated, and the unit is converted into millimeters according to the ratio of 1:50 (1 mm = 50 px).

The effective defocusing contact range reflects the situation, in which the pupil encloses the treatment zone. Specifically, the location of the defocusing zone was calculated at first. The intersection of the pupil and the treatment zone was subtracted from the pupil. Then, the image was binarized (the function of binarization is to keep the foreground part of the segmented image unique in pixel values) and the boundary range between the defocused zone and the pupil boundary was calculated using the four-neighborhood algorithm, which is the effective contact value. The proportion of the effective contact value in the whole pupil boundary was calculated using the formula shown in Eq. 7, where EDCR is the effective defocusing contact range, ECV is the effective contact value, and C is the length of the pupil boundary.


(7) $$\begin{array}{l} \text{EDCR}=\frac{\text{ECV}}{\text{C}} \end{array}$$

### 2.6 Evaluation criteria for corneal topography type

After calculating the decentration and effective defocusing contact range mentioned earlier, we incorporated them into the evaluation criteria provided by the optometrist to evaluate the corneal topography. The evaluation criteria are as follows:

Class I: 0 mm ≤ decentration ≤ 1 mm, effective defocusing contact range > 3/4.
Class II: 0 mm ≤ decentration ≤ 1 mm, effective defocusing contact range is 1/4–3/4.
Class III: decentration > 1 mm.
Class IV: decentration ≤ 0.5 mm, effective defocusing contact range < 1/4.

There is no absolute difference between “good” and “bad” topography. For example, for most younger myopic children, optometrists prefer the corneal topography to be class I after fitting the OK lens. The small decentration of this type of topography could control axial elongation and correct refractive errors. While for older adolescents, the myopia progression reaches a stable state, the class IV of corneal topography is more appropriate. Therefore, the corneal topography type evaluation method described in this article can effectively analyze the corneal topography of various patients to facilitate optometrists' development of personalized laboratory fitting programs for patients.

## 3 Data analysis

Data analysis was performed using third-party libraries Matplotlib 2.2.3 and scikit-learn 0.6.1. For semantic segmentation of important medical zones, the scikit-learn library was used to calculate the precision, recall, F1-score, and IoU. Finally, we compared the number and accuracy of various types of corneal topographies with the labels given by the optometrist and counted the relationship between them and the 1-year axial elongation.

## 4 Results and analysis

### 4.1 Results of the treatment zone and pupil segmentation

In the past, when optometrists identified the treatment zone of the corneal topography, some errors might have occurred due to human subjectivity or long-term work. Table 1 shows the segmentation accuracy of the deep learning model through a number of evaluation indicators. It could be seen that the results of the model are very close to the human annotation results, and the performance of U-Net, U-Net++, and U-Net3+ is fairly comparable. However, U-Net exhibits a lower model complexity, implying that we could expedite the training and prediction processes. Furthermore, U-Net++ and U-Net3+, owing to their increased model complexity, were more prone to overfitting. In addition, considering model deployment, the lightweight and simplicity of U-Net make it more amenable to practical applications. Our research has already been applied at the Tianjin Eye Hospital Optometry Center, so selecting U-Net has facilitated the simplification of the deployment and maintenance processes. In summary, the U-Net model, due to its shorter training times, reduced susceptibility to overfitting, and ease of deployment, emerged as our preferred choice. Both spatial attention and channel attention were also attempted in our study in an effort to optimize the performance of the model. However, there was no observed improvement in the segmentation results. The U-Net model trained by CE loss was chosen (36) and evaluated from the aspects of precision, recall, F1-score, and IoU. To obtain optimal segmentation results, U-Net++ and U-Net3+ were used to carry out experiments. The results showed that U-Net3+ has the best segmentation performance. We thought it was mainly because U-Net3+ uses multiple short connections instead of long connections, which maximizes the semantic information of the original image. In addition, the deep learning model only took 0.06 s to segment the pupil and the treatment zone in the corneal topography. In conclusion, the deep learning model had faster processing speed and higher accuracy in the semantic segmentation of important medical zones.

**Table 1:** Segmentation results of the U-net, U-Net++, and U-Net3+ models.

**Network structure**	**Medical zone**	**Precision**	**Recall**	**F1-score**	**IoU**
U-Net	Pupil	0.9688	0.9723	0.9689	0.9412
		Treatment zone	0.9499	0.9308	0.9396	0.8802
U-Net++	Pupil	0.9687	0.9728	0.9699	0.9429
		Treatment zone	0.9512	0.9401	0.9423	0.8826
U-Net3+	Pupil	0.9771	0.9744	0.9712	0.9481
		Treatment zone	0.9587	0.9378	0.9459	0.8901

To further demonstrate the stability of the model, a K-fold cross-validation method was used to divide the dataset into 10 parts to train the model (*k* = 10). The method of dividing the dataset into 10 parts to train the model is to randomly divide the dataset into nine parts for each experiment as the training set and the other part as the validation set. The validation set for each experiment is different, and the entire experiment is repeated 10 times to ensure the robust of the model. As shown in Table 2, the results of the 10 experiments showed slight differences due to different datasets, but the overall performance was good. The F1-scores of the pupil and treatment zone were the highest at 0.9707 and 0.9409, respectively.

**Table 2:** K-fold cross-validation (*K* = 10).

	**Precision**		**Recall**		**F1-score**		**IoU**	
	**Pupil**	**Treatment zone**	**Pupil**	**Treatment zone**	**Pupil**	**Treatment zone**	**Pupil**	**Treatment zone**
1	0.9694	0.9487	0.9694	0.9308	0.9687	0.9396	0.9409	0.8808
2	0.9699	0.9505	0.9699	0.9312	0.9677	0.9409	0.9401	0.8812
3	0.9678	0.9501	0.9716	0.9277	0.9697	0.9389	0.9397	0.8804
4	0.9681	0.9497	0.9669	0.9287	0.9675	0.9392	0.9412	0.8809
5	0.9687	0.9485	0.9727	0.9274	0.9707	0.9379	0.9417	0.8797
6	0.9701	0.9479	0.9661	0.9273	0.9681	0.9376	0.9411	0.8795
7	0.9669	0.9497	0.9651	0.9301	0.9660	0.9399	0.9414	0.8819
8	0.9677	0.9499	0.9725	0.9305	0.9701	0.9402	0.9415	0.8821
9	0.9692	0.9489	0.9630	0.9275	0.9661	0.9381	0.9406	0.8801
10	0.9685	0.9490	0.9719	0.9310	0.9702	0.9400	0.9415	0.8821

### 4.2 Calculation results of the medical indicators

#### 4.2.1 Decentration calculation results

The results are shown in Supplementary Figure S1f, where dark gray is the contour of the treatment zone, light gray is the contour of the pupil, and the black straight line is the decentration. At the same time, special circumstances need to be considered (the results are shown in Supplementary Figure S2). There may be noise after the deep learning segmentation model segments the medical zone. It was necessary to calculate the maximum value of the zone under each contour in the segmentation image as the desired medical zone and understand other zones as noise for removal.

#### 4.2.2 Calculation results of effective defocusing contact range

The effective defocusing contact range indicated how much the treatment zone is inside the pupil. The results of each step were as follows. (1) The original figure (Supplementary Figure S3a) was transferred into the trained U-Net3+ model to segment the pupil (Supplementary Figure S3b) and the treatment zone (Supplementary Figure S3c). (2) The pupil was used to intersect with the treatment zone (Supplementary Figure S3d). (3) The defocused zone was obtained by subtracting this intersection from the pupil (Supplementary Figure S3e). (4) The pupil and the defocusing zone were binarized (Supplementary Figure S3g). (5) The four-neighborhood algorithm was used to detect the boundary points of the pupil and the defocusing zone. The proportion of their coincident boundary points (purple line) to the total circumference of the pupil was calculated, which was the effective defocusing contact range (Supplementary Figure S3h).

### 4.3 Research on the results of corneal topography type assessment and axial elongation

Following the calculation of the decentration and effective defocusing contact range, the corneal topography was evaluated according to the recommendation suggested by the Tianjin Eye Hospital Optometry Center. The relationship between corneal topography and axial elongation was analyzed to verify the suitability of different corneal topographies for patients of different ages. As the rate of axial elongation in older adolescents is slower, optometrists need to mainly consider visual quality when fitting OK lenses. The treatment zone of the class IV corneal topography in Table 3 was relatively large, and the decentration was relatively small, resulting in better visual quality in patients. As for younger children, they were in a period of rapid myopia development, and more attention should be paid to controlling the growth rate of the axial length during the fitting of the OK lens. However, excessive decentration might impact the effects of the corneal reshaping of the OK lens. In general, class I or class II of corneal topography might be more suitable for younger children.

**Table 3:** Statistics of corneal topography and axial elongation.

	**Class 1**	**Class 2**	**Class 3**	**Class 4**
Number of samples	933	1,167	375	129
Accuracy	99%	98%	99%	99%
1-year axial elongation	0.2	0.13	0.12	0.28
Standard deviation of axial length	0.21	0.17	0.07	0.11

### 4.4 Efficiency of machine learning

This experiment was developed under the Linux system, and the video card was Nvidia 2080Ti. The statistical analysis of 10 program runs showed that the average running time of the method described in this article is 2.2 s [Intel Core (TM) i7-9700K CPU @ 3.6 GHz]. It saved manpower and time compared with the optometrist, who spends more than half a minute to analyze the results.

## 5 Discussion

### 5.1 The need for models

Usually, traditional curvature information from corneal topography is used to determine the parameters of OK lenses (37). After a series of fluorescence evaluations and several trials of lenses, we can obtain lenses that are suitable for patients. This process depends on the experience of the optometrist and is very time-consuming. It was worth noting that the risk of corneal damage and cross-infection may be increased during the fitting process of an OK lens (38, 39). Therefore, clinicians hope to reduce the number of trial lenses as much as possible.

In this study, an effective method for corneal topography type evaluation was proposed, which can accurately calculate various medical indicators. Optometrists could set the best values for important medical indicators for different patients. At this time, the system would classify the corneal topography type, calculate the decentration, select effective defocusing contact range, etc. The optometrists then could quickly select the optimal OK lens for the patients and reduce the number of tests required, which plays an important role in preventing and controlling the risk of cross-infection.

### 5.2 The advantage of the model

A deep learning model could more easily solve problems that are difficult to describe with objective criteria, especially for the segmentation of treatment zones. The treatment zone is originally referred to as the zone in the corneal topography where the diopter of the anterior surface of the cornea changes < 0.50 D compared with that at the apex of the cornea (40). However, due to the uncertainty of the corneal state during the fitting of the OK lens, the treatment zone of some patients was not a relatively complete closed zone, but the optometrist hoped to get a complete zone for further analysis. Therefore, it is difficult to identify the treatment zone of all people with a fixed diopter value. At this time, the semantic segmentation model based on deep learning could better solve this problem. After extracting features from many corneal topographies, the model could understand which part of the treatment zone corresponds to it and segment it.

Previous studies only identified the treatment zone (41). As far as we know, there was no objective evaluation method to classify the corneal topography type according to the decentration and effective defocusing contact range. These indicators were usually calculated through professional medical guidance. The current method proposed by us could help optometrists carry out customized fittings according to the different ages of patients so that they could finally present the most favorable corneal topography type for them. The OK lens could be replaced immediately to avoid a decrease in visual quality if the type of corneal topography was mismatched with age or the value of medical indicators was abnormal during each review. It eliminated the risks that human subjectivity may bring to patients and enhanced the reliability of diagnosis.

In addition, it takes 1–2 min for the optometrist to evaluate the corneal topography results in the usual way and input them into the computer, and there is a chance of interference due to external factors in the process. However, the error caused by subjectivity can be avoided based on the method described in this article, and the data calculation and acquisition efficiency could be effectively improved.

### 5.3 Model performance

It could be seen that the results of the model are very close to the human annotation results, and the performance of U-Net, U-Net++, and U-Net3+ is fairly comparable. However, U-Net exhibits a lower model complexity, implying that we could expedite the training and prediction processes. Furthermore, U-Net++ and U-Net3+, owing to their increased model complexity, were more prone to overfitting. In addition, considering model deployment, the lightweight and simplicity of U-Net make it more amenable to practical applications. Our research has already been applied at the Tianjin Eye Hospital Optometry Center, so selecting U-Net has facilitated the simplification of the deployment and maintenance processes. In summary, the U-Net model, due to its shorter training times, reduced susceptibility to overfitting, and ease of deployment, emerged as our preferred choice. In our study, spatial attention and channel attention were also attempted in an effort to optimize the performance of the model. However, there was no observed improvement in the segmentation results. The U-Net model trained by CE loss was chosen, and the model was evaluated from the aspects of precision, recall, F1-score, and IoU. In addition, the deep learning model only took 0.06 s to segment the pupil and the treatment zone in the corneal topography. In conclusion, the deep learning model had faster processing speed and higher accuracy in the semantic segmentation of important medical zones.

### 5.4 Limitations

A larger sample size could optimize the deep learning model to avoid overfitting. This method can effectively improve the segmentation accuracy of the pupil and treatment zone, calculate medical evaluation indicators more accurately, and analyze and evaluate the type of corneal topography more reasonably, which can effectively improve the efficiency of lens fitting. In addition, this study is based on the images collected by Tomey corneal topography. Images generated by other brands of corneal topographers can also use the method described in this article to grade the treatment effect of corneal topographers. However, it is necessary to retrain the segmentation model of the treatment zone and pupil and fine-tune the subsequent image processing algorithm.

## 6 Conclusion

This study proposed a novel method to evaluate corneal topography based on deep learning. This process formulated corresponding medical indicators and accurately calculated them according to the analysis of images. At the same time, it reduced the pressure of time and manpower on optometrists and assisted optometrists in follow-up treatment. This method provided a new idea for the analysis of corneal topography, and the corneal topography of other brands could be analyzed through simple changes, thus guiding the intelligent fitting of OK lenses.

## Data availability statement

The raw data supporting the conclusions of this article will be made available by the authors, without undue reservation.

## Ethics statement

This study was approved by the Ethics Committee of Tianjin Eye Hospital Optometric Center (ID 2023003) and adhered to the tenets of the Declaration of Helsinki. The studies were conducted in accordance with the local legislation and institutional requirements. Written informed consent for participation was not required from the participants or the participants' legal guardians/next of kin in accordance with the national legislation and institutional requirements.

## Author contributions

SX: Data curation, Formal analysis, Writing – original draft. XY: Data curation, Formal analysis, Writing – original draft. SZ: Data curation, Formal analysis, Writing – original draft. XZ: Formal analysis, Writing – original draft. FZ: Formal analysis, Writing – original draft. YL: Formal analysis, Writing – review & editing. HZ: Conceptualization, Funding acquisition, Methodology, Visualization, Writing – review & editing. LL: Funding acquisition, Validation, Visualization, Writing – review & editing. QY: Conceptualization, Project administration, Supervision, Visualization, Writing – review & editing.

## References

[1] Baird PN Saw SM Lanca C Guggenheim JA Smith EL Zhou X. Myopia. Nat Rev Dis Primers. (2020) 6:1–20. 10.1038/s41572-020-00231-4 33328468

[2] Grzybowski A Kanclerz P Tsubota K Lanca C Saw SM. A review on the epidemiology of myopia in school children worldwide. BMC Ophthalmol. (2020) 20:1–11. 10.1186/s12886-019-1220-0 31937276 PMC6961361

[3] Dong L Kang YK Li Y Wei W Jonas JB. Prevalence and time trends of myopia in children and adolescents in China: a systemic review and meta-analysis. Retina. (2020) 40:399–411. 10.1097/IAE.0000000000002590 31259808

[4] Naidoo KS Fricke TR Frick KD Jong M Naduvilath T Resnikoff S. Potential lost productivity resulting from the global burden of myopia: systematic review, meta-analysis, and modeling. Ophthalmology. (2019) 126:338–46. 10.1016/j.ophtha.2018.10.029 30342076

[5] Walline JJ Greiner KL McVey ME Jonas-Jordan EA. Multifocal contact lens myopia control. Optometry and Vision Science. (2013) 90:1207–14. 10.1097/OPX.0000000000000036 24061152

[6] Varnas S Gu X Metcalfe A. Bayesian meta-analysis of myopia control with multifocal lenses. J Clin Med. (2021) 10:730. 10.3390/jcm10040730 33673218 PMC7917905

[7] van Hees K Steenbekkers G Beerten R. Evaluation of children in the Netherlands fitted with custom made ortho-k contact lenses. Contact Lens Anterior Eye. (2022) 45:1. 10.1016/j.clae.2022.101647

[8] Jiang J Lian L Wang F Duncan M. Clinical effect of toric ortho-K lens. Invest Ophthalmol Vis Sci. (2018) 59:1744–1744. 10.1167/iovs.18-25067

[9] Tang WC Leung M Wong ACK To C Lam C. Optical interventions for myopia control. Updates on Myopia: Clini Perspect. (2020) 202:289–305. 10.1007/978-981-13-8491-2_1437740053

[10] Hashemi H Heydarian S Hooshmand E Saatchi M Yekta A Aghamirsalim M. The prevalence and risk factors for keratoconus: a systematic review and meta-analysis. Cornea. (2020) 39:263–70. 10.1097/ICO.0000000000002150 31498247

[11] Chou CC Huang YC Tsai YY Lin JM Chen W Lin H. Changes in corneal curvature after fitting the orthokeratology lens. Taiwan J Ophthalmol. (2013) 3:156–9. 10.1016/j.tjo.2013.10.001

[12] Kanclerz P Khoramnia R Wang X. Current developments in corneal topography and tomography. Diagnostics. (2021) 11:1466. 10.3390/diagnostics11081466 34441401 PMC8392046

[13] Schiano-Lomoriello D Bono V Abicca I. Repeatability of anterior segment measurements by optical coherence tomography combined with Placido disk corneal topography in eyes with keratoconus. Sci Rep. (2020) 10:1–6. 10.1038/s41598-020-57926-7 31980662 PMC6981210

[14] Fan Y Yu Z Tang T Liu X Xu Q Peng Z. Machine learning algorithm improves accuracy of ortho-K lens fitting in vision shaping treatment. Cont Lens Anterior Eye. (2022) 45:101474. 10.1016/j.clae.2021.101474 34301476

[15] Wei RH Lim L Chan WK Tan D. Evaluation of Orbscan II corneal topography in individuals with myopia. Ophthalmology. (2006) 113:177–83. 10.1016/j.ophtha.2005.11.004 16458090

[16] Calvo-Sanz JA Ruiz-Alcocer J Sánchez-Tena MA. Intraocular lens bicylindric power calculation method: Using both flat and steep K readings to improve intraocular lens power prediction. Eur J Ophthalmol. (2018) 28:559–65. 10.1177/1120672117754170 29566552

[17] Dao CL Kok JH Brinkman CJ Mill CJ. Corneal eccentricity as a tool for the diagnosis of keratoconus. Cornea. (1994) 13:339–44. 10.1097/00003226-199407000-00009 7924334

[18] Maseedupally VK Gifford P Lum E Sidawi D Wang B Swarbick HA. Treatment zone decentration during orthokeratology on eyes with corneal toricity. Optomet Vision Sci. (2016) 93:1101–11. 10.1097/OPX.0000000000000896 27254811

[19] Lin W Gu T Bi H Zhang B Wei R. The treatment zone decentration and corneal refractive profile changes in children undergoing orthokeratology treatment. BMC Ophthalmol. (2022) 22:1–9. 10.1186/s12886-022-02396-w 35436922 PMC9016930

[20] Sun L Li ZX Chen Y He Z Song H. The effect of orthokeratology treatment zone decentration on myopia progression. BMC Ophthalmol. (2022) 22:1–7. 10.1186/s12886-022-02310-4 35164702 PMC8845411

[21] Tsai YY Lin JM. Ablation centration after active eye-tracker-assisted photorefractive keratectomy and laser in situ keratomileusis. J Cataract Refract Surg. (2000) 26:28–34. 10.1016/S0886-3350(99)00328-4 10646143

[22] Kuo BI Chang WY Liao TS Liu F Liu H Chu H. Keratoconus screening based on deep learning approach of corneal topography. Transl Vis Sci Technol. (2020) 9:53–53. 10.1167/tvst.9.2.53 33062398 PMC7533740

[23] Shanthi S Aruljyothi L Balasundaram MB Janakiraman A Nirmaladevi K Pyingkodi M. Artificial intelligence applications in different imaging modalities for corneal topography. Surv Ophthalmol. (2022) 67:801–16. 10.1016/j.survophthal.2021.08.004 34450134

[24] Liu W Wang Z Liu X Zeng N Liu Z Alsaadi FE. A survey of deep neural network architectures and their applications. Neurocomputing. (2017) 234:11–26. 10.1016/j.neucom.2016.12.038

[25] Gao HB Pan ZG Shen MX Lu F Li H Zhang X. KeratoScreen: early keratoconus classification with zernike polynomial using deep learning. Cornea. (2022) 41:1158–65. 10.1097/ICO.0000000000003038 35543584

[26] Hao S Zhou Y Guo Y. A brief survey on semantic segmentation with deep learning. Neurocomputing. (2020) 406:302–21. 10.1016/j.neucom.2019.11.118

[27] Wang P Chen P Yuan Y Liu D Huang Z Hou X. Understanding convolution for semantic segmentation.2018 IEEE winter conference on applications of computer vision (WACV). IEEE. (2018) 2018:1451–60. 10.1109/WACV.2018.00163

[28] Racine L Wang L Koch DD. Size of corneal topographic treatment zone: comparison of standard and customized myopic laser in situ keratomileusis. Am J Ophthalmol. (2006) 142:227–32. 10.1016/j.ajo.2006.03.023 16876501

[29] Hilmi MR Kamal KM Azemin MZC Ithnin MH Mustafa MS Ahmad N. Corneal curvature measurements utilizing a new swept-source optical coherence tomography Tomey OA-2000^®^ and comparison with IOL Master^®^ 500 in pterygium patients. Sains Medika. (2018) 9:11–7. 10.30659/sainsmed.v9i1.2918

[30] Venkateswaran N Galor A Wang J Karp CL. Optical coherence tomography for ocular surface and corneal diseases: a review. Eye and Vision. (2018) 5:1–11. 10.1186/s40662-018-0107-0 29942817 PMC5996489

[31] Barkau RL. UNET: One-dimensional unsteady flow through a full network of open channels. User's manual. In:Oktay O Schlemper J Folgoc LL, editors. Attention u-Net: Learning Where to Look for the Pancreas. Davis CA: Hydrologic Engineering Center. (1996).

[32] Smith EL. Optical treatment strategies to slow myopia progression: effects of the visual extent of the optical treatment zone. Exp Eye Res. (2013) 114:77–88. 10.1016/j.exer.2012.11.019 23290590 PMC3624048

[33] Russell BC Torralba A Murphy KP Freeman W. LabelMe: a database and web-based tool for image annotation. Int J Comput Vis. (2008) 77:157–73. 10.1007/s11263-007-0090-8

[34] Da K. Adam: A method for stochastic optimization. arXiv preprint arXiv:1412.6980. 10.48550/arXiv.1412.6980

[35] Bradski G. The openCV library. Dobb's J. (2000) 25:120–3.

[36] De Boer PT Kroese DP Mannor S Rubinstein RY. A tutorial on the cross-entropy method. Ann Operat Res. (2005) 134:19–67. 10.1007/s10479-005-5724-z

[37] Li C Zeng L Zhou J Wang B Chen Z. To achieve a bullseye: factors related to corneal refractive therapy orthokeratology lens toricity. J Clin Med. (2022) 11:5635. 10.3390/jcm11195635 36233502 PMC9572783

[38] Hogan RN. Potential for transmission of prion disease by contact lenses: an assessment of risk. Eye Contact Lens. (2003) 29:44–8. 10.1097/00140068-200301001-00013 12772730

[39] Luai AI Wisam S Sana J. Antimicrobial susceptibility of bacterial isolates from the conjunctiva, storage cases and mobile phones of university students using contact lenses. Cont Lens Anter Eye. (2021) 44:62–6. 10.1016/j.clae.2019.10.139 31722813

[40] Camellin M Arba Mosquera S. Aspheric optical zones: the treatment zone with the SCHWIND AMARIS. J Refract Surg. (2011) 27:135–46. 10.3928/1081597X-20100428-03 20481411

[41] Tang Y Chen Z Wang W Wen L Zhou L Wang M. A Deep learning–based framework for accurate evaluation of corneal treatment zone after orthokeratology. Transl Vis Sci Technol. (2021) 10:21–21. 10.1167/tvst.10.14.21 34932118 PMC8709934

