# Supplementary material for: Evaluation of the corneal topography based on deep learning
Source: Front Med (Lausanne). 2024 Jan 4;10:1264659. doi: 10.3389/fmed.2023.1264659 (PMC10794654; doi:10.3389/fmed.2023.1264659)
Supplement: Supplementary file 1 [file Image_1.pdf]

Supplementary Material

Evaluation of the corneal topography based on deep learning

Authors:
Shuai Xu[a,#], Xiaoyan Yang[b,c,d,#], Shuxian Zhang[b,c,d], Xuan Zheng[a], Fang Zheng[a], Yin Liu[e], Hanyu Zhang[e*], Lihua Li[b,c,d,*] Qing Ye[a,*]
Author affiliations
a. Key Laboratory of Weak-Light Nonlinear Photonics, Ministry of Education, School of Physics and TEDA Applied Physics, Nankai University, Tianjin 300071, China
b. Tianjin Eye Hospital, Tianjin, China
c. Tianjin Key Lab of Ophthalmology and Visual Science, Tianjin, China
d. Nankai University Affiliated Eye Hospital, Tianjin, China
e. Eye Hospital Optometric Center, Tianjin, China
f. School of Medicine, Nankai University, Tianjin 300071, China.

This PDF file includes:

Fig.S1 Decentration calculation process.

Fig.S2 Noise points in the processing division diagram.

Fig.S3 Calculation flow of effective defocusing contact range.

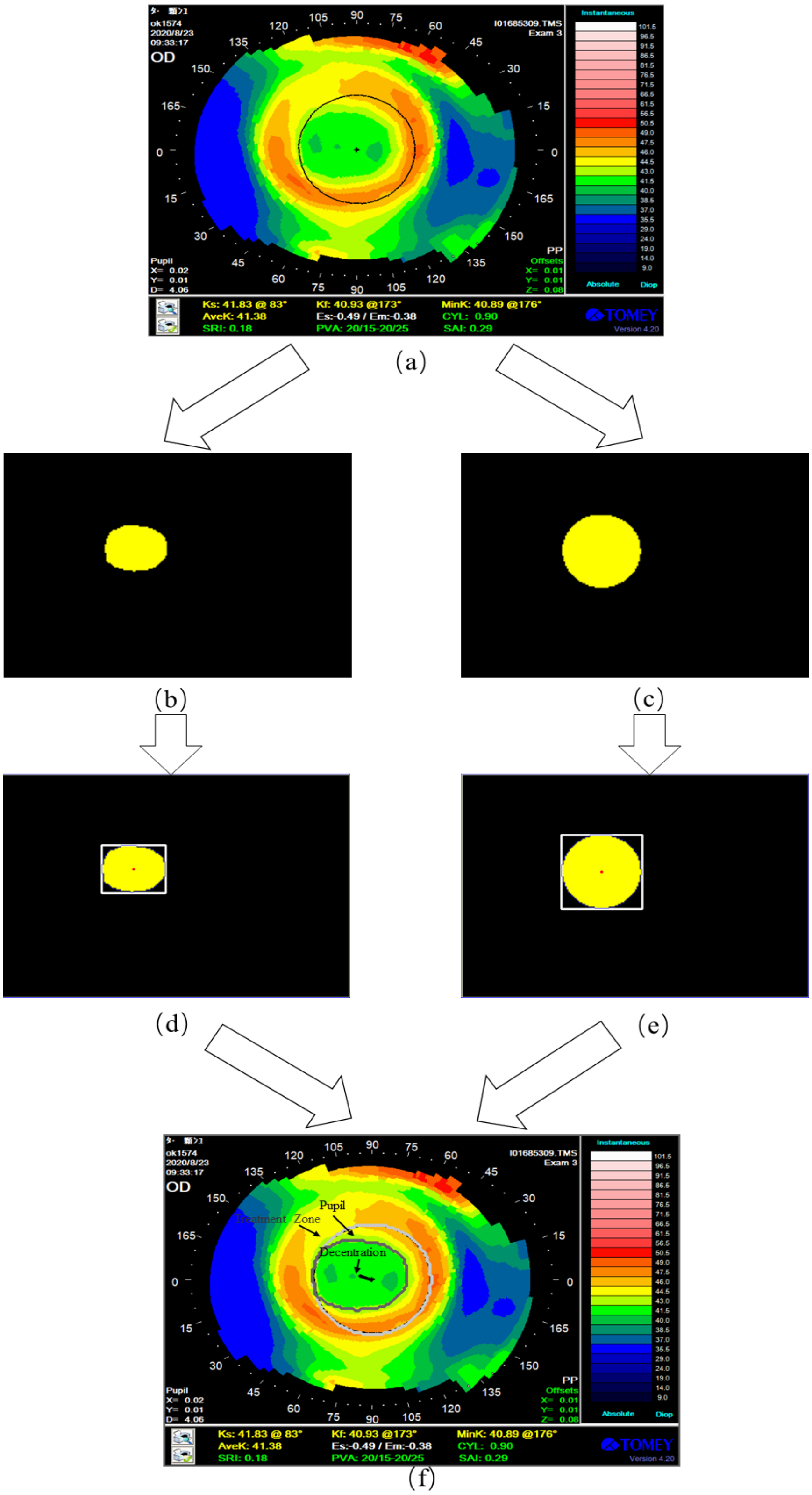

Figure S1. Decentration calculation process. (a) Original picture (b) Treatment zone after segmentation (c) Pupils after segmentation (d) Positioning the center point of treatment zone (e) Positioning the center point of pupil (f) Effect picture

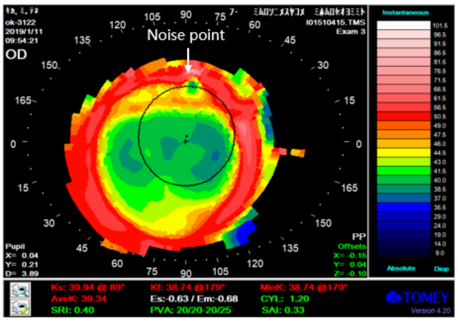

(a)

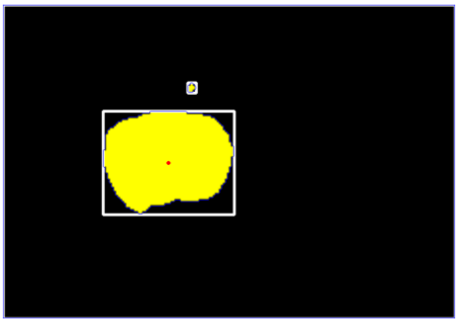

(b)

Figure S2. Noise Point Processing (a) Original Figure (b) Noise Points in the Processing Division Diagram.

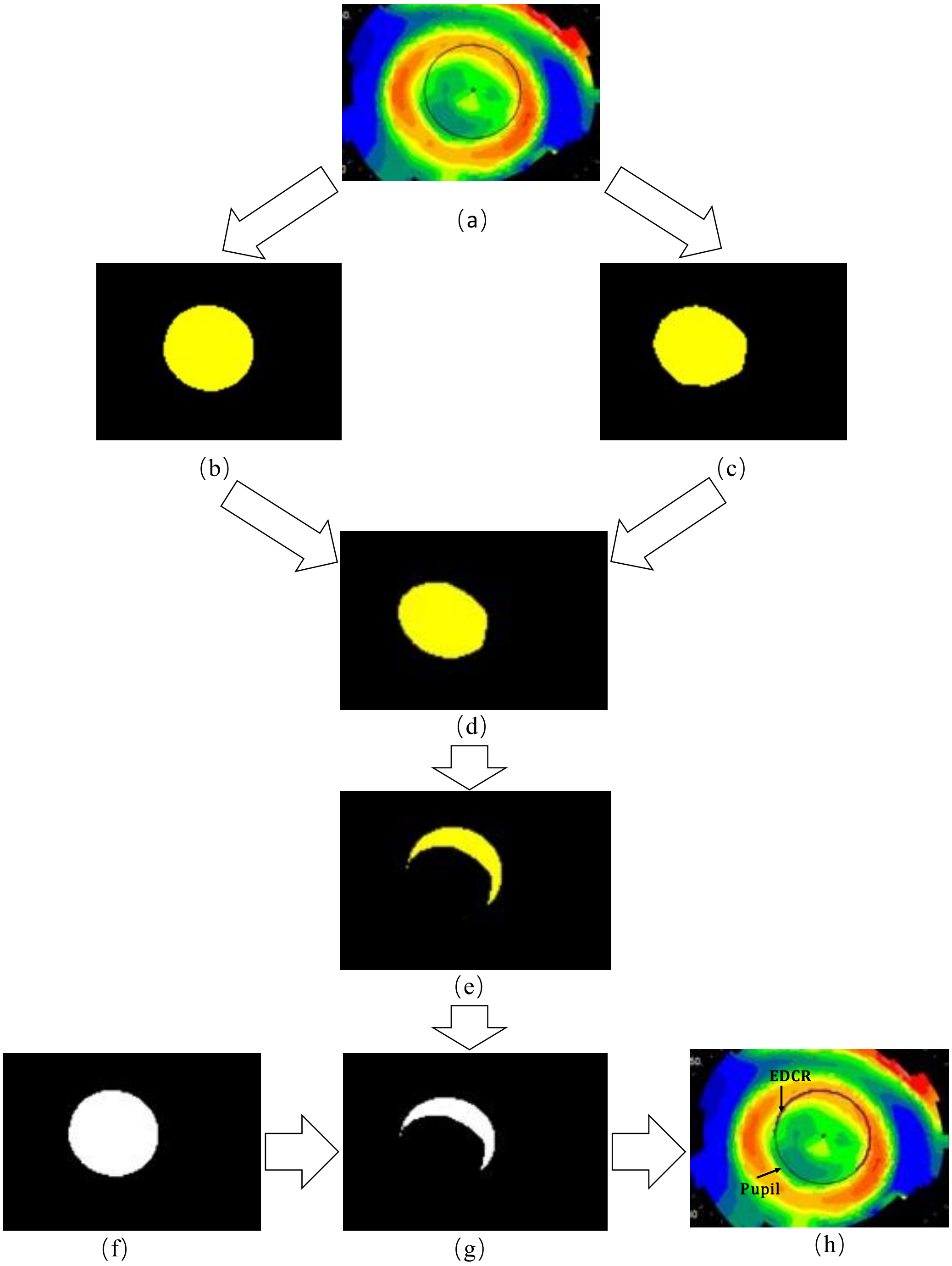

Figure S3. Calculation flow of effective defocusing contact range. (a) Original picture (b) segmented pupil (c) segmented treatment zone (d) intersection of pupil and treatment zone (e) defocused zone (f) binarization of pupil (g) binarization of defocused zone (h) effect picture
